# Myocardial Perfusion in ST-Segment Elevation Myocardial Infarction Patients After Percutaneous Coronary Intervention: Influencing Factors and Intervention Strategies

**DOI:** 10.7759/cureus.42841

**Published:** 2023-08-02

**Authors:** Nan Tang, Xuejin Chen, Kangming Li, Haoran Li, Chunmei Qi

**Affiliations:** 1 Department of Cardiology, The Second Affiliated Hospital of Xuzhou Medical University, Xuzhou, CHN

**Keywords:** intervention strategies, risk factors, myocardial perfusion, percutaneous coronary intervention, st-segment elevation myocardial infarction

## Abstract

Aim

We aim to explore the factors influencing myocardial perfusion in patients with acute ST-segment elevation myocardial infarction (STEMI) after primary percutaneous coronary intervention (PPCI) and evaluate the effects of different intervention strategies on myocardial perfusion improvement.

Methods

A retrospective analysis was conducted on 300 patients with STEMI who underwent primary percutaneous coronary intervention (PPCI) at our hospital between January 2020 and December 2022. Based on post-procedural coronary angiography results using the thrombolysis in myocardial infarction (TIMI) blood flow grade and myocardial blush grade (MBG), patients were categorized into two groups: the normal perfusion group (TIMI grade 3 or MBG 2-3, n=180) and the impaired perfusion group (TIMI grades 0-2 or MBG 0-1, n=120). The impaired perfusion group was further divided using a random number table into the thrombus aspiration-only group (control group, n=60) and the thrombus aspiration combined with nicorandil group (nicorandil group, n=60). A 1:1 propensity score matching method was employed to adjust for baseline characteristics between the groups. Clinical characteristics, hematological parameters, coronary lesion features, and percutaneous coronary intervention (PCI) technical parameters were compared between the matched groups. Additionally, a multivariate logistic regression analysis was performed to identify independent risk factors influencing myocardial perfusion. Furthermore, the post-procedural myocardial perfusion, cardiac function, and clinical prognosis were compared between the control and nicorandil groups.

Results

After matching, the baseline characteristics of the two groups were compared. The impaired perfusion group had older age, higher proportion of male patients, higher rates of diabetes and hypertension, longer time from symptom onset to balloon dilation, higher peak cardiac troponin I (cTnI) levels, higher proportion of left main or multivessel involvement, heavier coronary lesion burden, and lower balloon inflation pressure (P<0.05). Multivariate logistic regression analysis revealed that age of ≥65 years (odds ratio {OR}=2.34, 95% confidence interval {CI}=1.23-4.46, P<0.01), time from symptom onset to balloon dilation of ≥6 hours (OR=3.12, 95% CI=1.67-5.83, P<0.01), peak cTnI level of ≥100 ng/mL (OR=4.27, 95% CI=2.18-8.36, P<0.01), left main or multivessel involvement (OR=2.86, 95% CI=1.51-5.41, P<0.01), and balloon inflation pressure of <8 atm (OR=3.45, 95% CI=1.79-6.65, P<0.01) were independent risk factors affecting myocardial perfusion. In the intervention analysis, the nicorandil group showed superior post-procedural TIMI blood flow grade, MBG, left ventricular ejection fraction (LVEF), and New York Heart Association (NYHA) functional classification compared to the control group (P<0.05). During a six-month follow-up, the nicorandil group had a lower incidence of major adverse cardiovascular events (MACE) compared to the control group (P<0.05).

Conclusion

Age, time from symptom onset to balloon dilation, peak cTnI level, extent of coronary artery lesions, and balloon inflation pressure were identified as independent risk factors affecting myocardial perfusion in STEMI patients after PCI. Compared to simple thrombus aspiration, thrombus aspiration combined with nicorandil demonstrated better improvement in myocardial perfusion, cardiac function, and clinical outcomes for patients with impaired perfusion.

## Introduction

ST-segment elevation myocardial infarction (STEMI) is the most severe type of acute coronary syndrome (ACS), characterized by the complete or near-complete occlusion of the coronary arteries, leading to myocardial ischemic necrosis and significantly endangering the patient's life and cardiac function[1]. Primary percutaneous coronary intervention (PPCI) is the preferred emergency treatment for STEMI patients, aiming to rapidly restore coronary artery blood flow, reduce myocardial damage, and improve prognosis [2]. However, not all STEMI patients treated with PPCI achieve optimal myocardial perfusion. Even after the successful reopening of the occluded vessel, the myocardium may still not receive effective perfusion, as indicated by the thrombolysis in myocardial infarction (TIMI) flow grade being less than 3, manifesting as slow, incomplete, or absent coronary blood flow. This phenomenon is known as "no reflow" (NR) and occurs in approximately 10%-30% of cases [3,4]. NR is closely associated with short-term and long-term adverse events, length of hospital stay, medical costs, and mortality rates in STEMI patients [5]. Therefore, studying the factors influencing myocardial perfusion after PPCI in STEMI patients and proposing corresponding intervention strategies are of great significance in improving treatment outcomes and quality of life.

Common methods used to handle high thrombus burden during PPCI include thrombus aspiration, intracoronary thrombolytic therapy, and excimer laser ablation [6]. Among them, residual thrombus fragments during thrombus aspiration may flow into the distal coronary artery, leading to microembolization [7,8]. Nicorandil, a novel adenosine triphosphate (ATP)-sensitive potassium channel opener, improves myocardial tissue-level perfusion and is recommended as the first-line drug for the treatment of microvascular disease according to guidelines [9]. However, there is still limited research on the local application of nicorandil through the thrombus aspiration catheter. Several studies have reported factors influencing myocardial perfusion after PPCI in STEMI patients, including patient-related factors, coronary lesion factors, and percutaneous coronary intervention (PCI) procedural factors [10]. Nevertheless, most of these studies are single-center or small-sample observational studies, with inconsistent results and lack of a systematic, comprehensive investigation, as well as targeted intervention strategies [11,12]. Therefore, the aim of this study is to conduct a retrospective analysis of STEMI patients admitted to our hospital in the past two years, to explore the factors influencing myocardial perfusion after PPCI, and to evaluate the effects of different intervention strategies on myocardial perfusion improvement.

## Materials and methods

Research participants

This study conducted a retrospective analysis of 300 STEMI patients who underwent PPCI treatment at our hospital from January 2020 to December 2022. Among them, there were 234 male and 66 female patients, with an age range of 45-85 years and a mean age of 64.5±10.2 years.

The inclusion criteria were based on the diagnostic criteria of "2019 Chinese Society of Cardiology (CSC) guidelines for the diagnosis and management of patients with ST-segment elevation myocardial infarction" [3]: (1) acute myocardial ischemia symptoms, such as squeezing chest pain located behind the sternum or in the precordial area, lasting for more than 10-20 minutes, possibly accompanied by radiating pain to the neck, shoulder, back, and left upper limb, in which some patients may experience symptoms such as nausea, vomiting, profuse sweating, dyspnea, or even syncope and the symptoms cannot be completely relieved by taking nitroglycerin; (2) electrocardiogram changes, characterized by upsloping ST-segment elevation (with some patients having pathological Q waves or decreased R waves) and corresponding mirror image ST-segment depression in the corresponding leads; and (3) serum and imaging evidence, dynamic elevation of cardiac injury markers, such as creatine kinase (CK) isoenzymes and troponin T, in the acute phase, and echocardiography showing segmental wall motion abnormalities.

The exclusion criteria were as follows: (1) severe liver or kidney dysfunction, bleeding disorders, or coagulation abnormalities; (2) other severe cardiac or systemic diseases, including a history of cardiac arrest or shock status, myocarditis, pericarditis, myocardial bridging, aortic dissection, and septic shock; (3) mechanical complications, history of previous myocardial infarction, or cardiac surgery; and (4) malignant tumors and severe anemia. This study was approved by the ethics committee of our hospital.

Study methods

The study collected general information on all patients at admission, including age, gender, body mass index (BMI), smoking history, alcohol consumption history, and family history. Clinical characteristics included time from onset to balloon dilation and the presence of diabetes, hypertension, hyperlipidemia, and atrial fibrillation. Blood indicators recorded were white blood cell (WBC) count, hemoglobin (Hb) level, platelet (PLT) count, creatine kinase-myocardial band (CK-MB) level, cardiac troponin I (cTnI) level, serum creatinine (SCr) level, blood urea nitrogen (BUN) level, total cholesterol (TC) level, triglyceride (TG) level, high-density lipoprotein cholesterol (HDL-C) level, low-density lipoprotein cholesterol (LDL-C) level, C-reactive protein (CRP) level, procalcitonin (PCT) level, D-dimer level, international normalized ratio (INR), activated partial thromboplastin time (APTT), and fibrinogen (FIB) level. For coronary angiography and PCI technical parameters, the data collected included the location and extent of coronary artery lesions, lesion complexity and burden, TIMI blood flow grade and myocardial blush grade (MBG), balloon dilation pressure and time, stent implantation site and number, and postoperative medication treatment. Postoperative cardiac function and clinical prognosis were assessed based on left ventricular ejection fraction (LVEF), New York Heart Association (NYHA) functional classification, and major adverse cardiovascular events (MACE).

The thrombolysis in myocardial infarction (TIMI) blood flow grade [13] was used to evaluate reperfusion: TIMI 0 indicates no reperfusion or the complete occlusion of the coronary artery without antegrade flow, TIMI 1 indicates partial perfusion with contrast passing the occluded site but the incomplete filling of the distal coronary artery, TIMI 2 indicates the complete filling of the distal coronary artery but slower contrast entry and clearance compared to normal coronary arteries, and TIMI 3 indicates normal coronary blood flow, with contrast filling and clearing rapidly in the distal vessel. Generally, TIMI 3 flow indicates normal perfusion, TIMI 2 indicates slow flow, and TIMI 0-1 indicates no reflow.

The myocardial blush grade (MBG) [3] was used to assess myocardial perfusion: MBG 0 indicates no blush, MBG 1 indicates minimal blush, MBG 2 indicates moderate blush but less than the non-infarct artery, and MBG 3 indicates normal blush, the same as the non-infarct artery. MBG 3 indicates complete myocardial reperfusion.

Major adverse cardiovascular events (MACE) [3] include cardiac death, non-fatal recurrent myocardial infarction, ischemia-driven target vessel revascularization, frequent angina attacks, and hospitalization for heart failure.

Grouping and intervention

All patients received 300 mg of aspirin and either 180 mg of ticagrelor or 300 mg of clopidogrel as antiplatelet therapy before emergency percutaneous coronary intervention (PCI). Prior to stent implantation, all culprit vessels with high thrombus burden were managed, which included thrombus aspiration using an aspiration catheter and/or intracoronary thrombolysis through the aspiration catheter. According to post-procedural coronary angiography's TIMI flow grade and myocardial blush grade (MBG), all patients were divided into the normal perfusion group (TIMI grade 3 or MBG grades 2-3, n=180) and the impaired perfusion group (TIMI grades 0-2 or MBG grades 0-1, n=120). The impaired perfusion group was further randomized into two subgroups: the pure thrombus aspiration group (control group, n=60) and the thrombus aspiration combined with nicorandil group (nicorandil group, n=60). Coronary angiography was performed using standard techniques to assess perfusion status. After guidewire passage to the distal part of the target lesion, thrombus was aspirated using the Thrombuster thrombus aspiration catheter (Terumo, Tokyo, Japan). In the nicorandil group, 4 mg of nicorandil was intracoronary injected through the thrombus aspiration catheter to the distal part of the target lesion.

Statistical analysis

This study used the Statistical Package for Social Sciences (SPSS) 26.0 (IBM SPSS Statistics, Armonk, NY) and EmpowerStats statistical software (X&Y Solutions Inc., Boston, MA) for data processing and analysis. Propensity score matching was employed to match the impaired perfusion group with the normal perfusion group. The matching factors included age, gender, BMI, smoking history, alcohol consumption history, family history, diabetes, hypertension, hyperlipidemia, atrial fibrillation, and time from onset to balloon dilation. The matching ratio was set at 1:1 for the impaired perfusion group to the normal perfusion group, with a caliper value set at 0.05. Continuous variables were presented as mean±standard deviation (x±s) or median (quartile range) based on whether they followed a normal distribution. Differences between the two groups were compared using t-tests for normally distributed data or Mann-Whitney U tests for non-normally distributed data. Categorical variables were presented as frequencies (percentages), and differences between the two groups were compared using chi-square tests or Fisher's exact tests. Multiple logistic regression analysis was performed to identify independent risk factors affecting myocardial perfusion and calculate the odds ratios (ORs) and 95% confidence intervals (CIs) for these factors. A significance level of P<0.05 was considered statistically significant for identifying differences between groups.

## Results

Comparison of general information to clinical characteristics before and after matching

The impaired perfusion group and the normal perfusion group were matched 1:1 based on 11 baseline indicators. After matching, there were 120 patients in both the impaired perfusion group and the normal perfusion group. The post-matching comparison showed no statistically significant differences in BMI, smoking history, alcohol consumption history, family history, hyperlipidemia, and atrial fibrillation between the two groups (P>0.05). However, there were statistically significant differences in age, gender, diabetes, hypertension, and time from onset to balloon dilation (P<0.05), as shown in Table 1.

**Table 1:** Comparison of general information to clinical characteristics before and after matching BMI: body mass index

Variables	Before Matching		P-value	After Matching		P-value
	Normal Perfusion Group (n=180)	Poor Perfusion Group (n=120)		Normal Perfusion Group (n=120)	Poor Perfusion Group (n=120)	
Age (years)	62.3±9.8	67.2±10.4	<0.05	62.1±9.6	67.2±10.4	<0.05
Gender (male/female)	132/48	102/18	<0.05	88/32	102/18	<0.05
BMI (kg/m^2^)	24.1±3.6	24.7±3.8	0.173	24.0±3.5	24.7±3.8	0.374
Smoking history, n (%)	72 (40.0)	54 (45.0)	0.321	48 (40)	54 (45.0)	0.232
Alcohol consumption history, n (%)	60 (33.3)	42 (35.0)	0.372	45 (37.5)	42 (35.0)	0.731
Family history, n (%)	36 (20.0)	24 (20.0)	0.456	26 (21.7)	24 (20.0)	0.572
Diabetes, n (%)	36 (20.0)	42 (35.0)	<0.05	25 (20.8)	42 (35.0)	<0.05
Hypertension, n (%)	72 (40.0)	66 (55.0)	<0.05	50 (41.7)	66 (55.0)	<0.05
Dyslipidemia, n (%)	48 (26.7)	36 (30.0)	0.261	33 (27.5)	36 (30.0)	0.512
Atrial fibrillation, n (%)	12 (6.7)	18 (15.0)	0.071	9 (7.5)	18 (15.0)	0.127
Time from symptom onset to balloon dilation (hours)	3.5 (2.5, 4.5)	5.5 (4, 7)	<0.05	3.4 (2.4, 4.4)	5.5 (4, 7)	<0.05

Comparison of blood indicators between the two groups after matching

There were no significant differences in WBC count, Hb, PLT count, SCr, BUN, TC level, TG, HDL-C, LDL-C, CRP, PCT, D-dimer, INR, APTT, FIB, and other parameters between the two groups (P>0.05). However, the impaired perfusion group had significantly higher peak levels of serum CK-MB and cTnI compared to the normal perfusion group, with statistical significance (P<0.05), as shown in Table 2.

**Table 2:** Comparison of blood indicators between the two groups after matching WBC, white blood cell; Hb, hemoglobin; PLT, platelet; CK-MB, creatine kinase-myocardial band; cTnI, cardiac troponin I; SCr, serum creatinine; BUN, blood urea nitrogen; TC, total cholesterol; TG, triglycerides; HDL-C, high-density lipoprotein cholesterol; LDL-C, low-density lipoprotein cholesterol; CRP, C-reactive protein; PCT, procalcitonin; INR, international normalized ratio; APTT, activated partial thromboplastin time; FIB, fibrinogen

Variables	Normal Perfusion Group (n=120)	Poor Perfusion Group (n=120)	P-value
WBC (×10^9^/L)	8.5±2.3	9.1±2.7	0.092
Hb (g/L)	136.3±16.6	134.2±18.4	0.342
PLT (×10^9^/L)	221.2±58.4	218.7±62.3	0.754
CK-MB (ng/mL)	36.2±12.4	48.5±16.7	<0.05
cTnI (ng/mL)	68.4±24.6	92.7±32.5	<0.05
SCr (μmol/L)	88.3±15.4	90.2±17.4	0.520
BUN (mmol/L)	5.6±1.8	5.8±2.1	0.573
TC (mmol/L)	4.8±1.2	4.9±1.3	0.325
TG (mmol/L)	1.7±0.8	1.8±0.9	0.624
HDL-C (mmol/L)	1.2±0.3	1.1±0.3	0.518
LDL-C (mmol/L)	2.9±0.9	3.0±1.0	0.463
CRP (mg/L)	12.3±6.7	13.2±7.4	0.526
PCT (ng/mL)	1.36±0.47	1.23±0.46	0.427
D-dimer (μg/mL)	1.8 (1.3, 2.4)	2.1 (1.6, 2.7)	0.192
INR	1.01±0.08	1.03±0.09	0.374
APTT(s)	28.5 (26.8, 30.2)	29.1 (27.4, 31)	0.263
FIB (g/L)	3.2 (2.9, 3.6)	3.3 (3.1, 3.7)	0.312

Comparison of coronary angiography to PCI technical parameters between the two groups after matching

There were no significant differences in the location of coronary artery lesions, lesion complexity, balloon dilation time, and the site and number of stent implantations between the two groups (P>0.05). However, the impaired perfusion group had a higher proportion of patients with the involvement of the left main or multiple vessels, a heavier burden of coronary artery plaques, and lower balloon dilation pressure compared to the normal perfusion group, with statistical significance (P<0.05), as shown in Table 3.

**Table 3:** Comparison of coronary angiography to PCI technical parameters between the two groups after matching LAD, left anterior descending; LCX, left circumflex; RCA, right coronary artery

Variables	Normal Perfusion Group (n=120)	Poor Perfusion Group (n=120)	P-value
Location of coronary artery lesions			0.093
LAD	64 (53.3%)	72 (60.0%)	
LCX	32 (26.7%)	30 (25.0%)	
RCA	24 (20.0%)	18 (15.0%)	
Involvement of the left main or multiple vessels	24 (20.0%)	54 (45.0%)	<0.05
Lesion complexity			0.085
A	16 (13.3%)	12 (10.0%)	
B1	32 (26.7%)	24 (20.0%)	
B2	40 (33.3%)	42 (35.0%)	
C	32(26.7%)	42 (35.0%)	
Burden of coronary artery plaques (%)	68.2±12.4	72.5±13.4	<0.05
Balloon dilation pressure (atm)	9.5±1.2	7.8±1.4	<0.05
Balloon dilation time (seconds)	30 (25, 35)	32 (28, 36)	0.061
Site of stent implantation (n)	1 (1, 2)	1 (1, 2)	0.173
Number of stents implanted (n)	1.2±0.4	1.3±0.5	0.154

Analysis of independent risk factors affecting myocardial perfusion

A multivariable logistic regression was conducted, utilizing general data, clinical traits, blood parameters, coronary angiography, and PCI technical parameters as predictors, while myocardial perfusion (normal/poor) served as the outcome variable. The objective of the analysis was to determine the variables that independently contribute to myocardial perfusion and compute their odds ratios (ORs) and 95% confidence intervals (CIs). Table 4 displays the findings. The myocardial perfusion is influenced by several independent risk factors, including being 65 years or older, having a time from symptom onset to balloon dilation of six hours or more, having a serum cTnI peak value of 100 ng/mL or higher, having the involvement of the left main or multiple vessels, and having a balloon dilation pressure below 8 atm.

**Table 4:** Analysis of independent risk factors affecting myocardial perfusion cTnI, cardiac troponin I; OR, odds ratio; CI, confidence interval

Factors	OR	95% CI	P-value
Age of ≥65 years	2.34	1.23-4.46	<0.01
Time from symptom onset to balloon dilation of ≥6 hours	3.12	1.67-5.83	<0.01
Serum cTnI peak value of ≥100 ng/mL	4.27	2.18-8.36	<0.01
Involvement of the left main or multiple vessels	2.86	1.51-5.41	<0.01
Balloon dilation pressure of <8 atm	3.45	1.79-6.65	<0.01

Comparison of intervention strategies

There were no statistically significant differences (P>0.05) in general information, clinical characteristics, blood indicators, coronary angiography, and PCI technical parameters between the control group and the nicorandil group, as shown in Table 5.

**Table 5:** Comparative analysis of general information and clinical characteristics among the two groups BMI, body mass index; CK-MB, creatine kinase-myocardial band; cTnI, cardiac troponin I; LAD, left anterior descending; LCX, left circumflex; RCA, right coronary artery

Variables	Control Group (n=60)	Nicorandil Group (n=60)	P-value
Age (years)	60.9±9.1	61.3±7.5	0.874
Gender (male/female)	36/24	39/21	1.625
BMI (kg/m^2^)	25.6±3.3	25.8±2.8	0.922
Smoking history, n (%)	24/36	27/33	0.852
Hypertension, n (%)	30/30	33/27	1.280
Diabetes, n (%)	12/48	15/45	0.852
Time from symptom onset to balloon dilation (hours)	5.3 (4.2, 6.6)	5.2 (3.9, 6.5)	0.892
CK-MB (ng/mL)	48.8±17.1	48.3±16.3	0.962
cTnI (ng/mL)	93.1±33.2	92.4±32.4	0.972
Location of coronary artery lesions			0.873
LAD	30 (50%)	33 (55%)	
LCX	18 (30%)	15 (25%)	
RCA	12 (20%)	12 (20%)	
Involvement of the left main or multiple vessels	33 (55%)	30 (50%)	0.680
Lesion complexity			0.795
A	3 (5%)	3 (5%)	
B1	15 (25%)	15 (25%)	
B2	27 (45%)	24 (40%)	
C	15 (25%)	18 (30%)	
Burden of coronary artery plaques (%)	73.2±13.9	72.9±13.5	0.975
Balloon dilation pressure (atm)	8.1±1.5	8±1.4	0.841
Balloon dilation time (seconds)	33 (29, 37)	32 (28, 36)	0.163
Site of stent implantation (n)	1 (1, 2)	1 (1, 2)	0.958
Number of stents implanted (n)	1.5±0.7	1.4±0.6	0.256

The comparison of intervention effects is presented in Table 6. In the nicorandil group, postoperative TIMI blood flow grade, MBG, LVEF, and NYHA functional classification were all superior to those in the control group (P<0.05). At the six-month follow-up, the occurrence rate of MACE (including cardiac death, non-fatal recurrent myocardial infarction, ischemia-driven target vessel revascularization, frequent angina attacks, and hospitalization for heart failure) was significantly lower in the nicorandil group compared to the control group (P<0.05).

**Table 6:** Comparison of intervention strategies TIMI, thrombolysis in myocardial infarction; MBG, myocardial blush grade (MBG); LVEF, left ventricular ejection fraction; NYHA, New York Heart Association; MACE, major adverse cardiac events

Variables	Control Group (n=60)	Nicorandil Group (n=60)	P-value
Post-procedural TIMI flow grade			<0.05
0	3 (5%)	0 (0%)	
1	9 (15%)	3 (5%)	
2	21 (35%)	9 (15%)	
3	27 (45%)	48 (80%)	
Post-procedural MBG grade			<0.05
0	6 (10%)	0 (0%)	
1	12 (20%)	3 (5%)	
2	18 (30%)	9 (15%)	
3	24 (40%)	48 (80%)	
Post-procedural LVEF (%)	54.7±6.7	60.5±5.5	<0.05
Post-procedural NYHA functional class			<0.05
I	24 (40%)	48 (80%)	
II	30 (50%)	12 (20%)	
III	6 (10%)	0 (0%)	
IV	0 (0%)	0 (0%)	
MACE occurrence rate at six-month follow-up	12 (20%)	3 (5%)	<0.05

## Discussion

According to the latest guidelines released by the European Society of Cardiology (ESC), primary percutaneous coronary intervention (PPCI) is considered the optimal choice for reperfusion in ST-segment elevation myocardial infarction (STEMI) patients [14]. In most cases, PPCI can achieve good myocardial reperfusion and improve patient prognosis. However, there is still a significant number of patients who experience impaired myocardial perfusion, and this is largely associated with microcirculation embolization in the distal coronary vessels, leading to the occurrence of the no reflow (NR) phenomenon. NR substantially increases the risk of major adverse cardiovascular events (MACE) after PPCI in STEMI patients. Studies have shown that the occurrence of NR during PPCI is related to various mechanisms, including thrombus or plaque embolization leading to microcirculation obstruction, microcirculation reperfusion injury, and microcirculation spasm in the coronary arteries [15]. Currently, there is no single effective method to prevent NR, although research suggests that drugs such as nitroprusside, verapamil, nicorandil, tirofiban, and adrenaline [16,17], as well as non-pharmacological approaches such as thrombus aspiration, distal protection devices, and MGuard stents, may help reduce or prevent NR [18,19]. However, the evidence in the field of evidence-based medicine is still inconclusive. Patients who experience NR after PPCI often have poorer prognoses, with higher mortality rates and rates of recurrent heart attacks, leading to adverse clinical and functional outcomes [20]. Therefore, analyzing the factors that contribute to impaired myocardial perfusion after PPCI in STEMI patients, evaluating effective strategies to prevent and treat NR, and enhancing the effectiveness of PPCI remain hot topics of discussion in the clinical setting.

The results of this study show that age, time from onset to balloon dilation, peak serum cTnI level, extent of coronary artery lesions, and balloon dilation pressure are independent risk factors affecting myocardial perfusion after PPCI in STEMI patients. These factors are related to the severity, duration, and extent of myocardial ischemia, as well as the degree of relief of the coronary arteries or microcirculation. Age is an important risk factor for cardiovascular disease, and elderly patients often have more comorbidities and more severe vascular sclerosis, leading to more severe and challenging myocardial ischemia and recovery [21]. The time from onset to balloon dilation is a crucial indicator reflecting the timing of myocardial reperfusion. Early reperfusion is generally considered more favorable, as delayed reperfusion can lead to irreversible myocardial cell necrosis and microcirculation disorders, increasing the risk of NR. Serum cTnI is a specific and highly sensitive biomarker of myocardial injury, and its peak value reflects the size and severity of myocardial infarction, which is also related to myocardial perfusion [22]. The extent of coronary artery lesions is an important indicator reflecting the range and severity of myocardial ischemia. The involvement of the left main or multiple vessels indicates a more extensive and deeper myocardial ischemia, which may complicate and make PCI treatment more difficult, possibly affecting myocardial perfusion [23]. Balloon dilation pressure is a significant parameter reflecting the quality and effectiveness of PCI procedures. Higher balloon dilation pressure ensures better stent apposition to the vessel wall and faster blood flow within the stent, which is favorable for improving myocardial perfusion. Therefore, in clinical practice, there should be increased attention to elderly STEMI patients, and a green channel should be established upon admission to promptly carry out PCI procedures, reducing the time to myocardial tissue reperfusion. Individualized surgical strategies and appropriate adjunctive medication should be adjusted based on each patient's condition to minimize the occurrence of no reflow during the procedure and ensure the effectiveness of PCI.

The use of manual thrombus aspiration catheters during PPCI can effectively remove thrombi or atheromatous plaque fragments causing impaired perfusion, thereby preventing the occurrence of coronary microcirculation disorders and distal vascular embolization in STEMI patients and reducing the risk of no reflow (NR) [24]. Although various drugs have been applied in clinical practice, the complex mechanisms leading to NR make the treatment challenging, and prevention remains the primary focus. The novel ATP-sensitive potassium channel opener, nicorandil, can open potassium channels and dilate the subepicardial coronary arteries and their microvessels, thereby reducing the occurrence of coronary microvascular dysfunction (CMD). Several recent studies investigating the use of nicorandil during PPCI for STEMI have shown that it can lower the incidence of NR, reduce major adverse cardiovascular events (MACE) (both short term and long term), and improve heart function [25,26]. Nicorandil can be administered before, during, or after PPCI, and thus far, no significant adverse reactions have been reported. It holds promise as a routine treatment to prevent NR.

The present study compared two methods: thrombus aspiration alone and thrombus aspiration combined with nicorandil. The nicorandil group showed significant improvements in post-PPCI myocardial perfusion, cardiac function, and clinical outcomes compared to the control group, suggesting that nicorandil can enhance myocardial tissue perfusion, limit the extent of myocardial infarction, and reduce reperfusion injury. Several small-sample studies have confirmed the preventive effect of nicorandil administered through various routes, such as oral administration before PPCI [27], intravenous infusion [28], and intracoronary injection into the target vessel [9], on no reflow during PPCI. This might be attributed to nicorandil's dual action as a potassium channel opener and a nitrate-like drug, which not only dilates coronary vessels, improves myocardial blood flow, and relieves myocardial ischemia but also exhibits beneficial effects in suppressing inflammatory responses, improving cardiac function, and inhibiting platelet aggregation. Mitochondrial ATP-sensitive potassium channels play a dominant role in local myocardial ischemic preconditioning and cardiac protection. When used for myocardial infarction treatment, nicorandil reduces the occurrence of coronary no reflow and exhibits ischemic preconditioning effects. Thrombus aspiration can clear thrombi or plaques within the coronary arteries, improving their patency or microcirculation. By removing large thrombi from the culprit vessel through thrombus aspiration and subsequently locally delivering thrombolytic drugs via aspiration catheters, a higher drug concentration can be achieved at the site of thrombus formation using a smaller drug dosage. This process weakens the fibrin structure and dissolves residual thrombi and tiny emboli that might cause distal embolization, leading to the more thorough removal of microthrombi and reducing the occurrence of no reflow, thereby improving microcirculation and perfusion. The results of this study align with expectations and provide clinicians with some effective intervention strategies to choose from.

This study is a small-scale retrospective investigation with a limited sample size, which may lead to biases that could affect the reliability and generalizability of the results. To ensure robustness and generalizability, further studies with a larger sample size are required for validation. Additionally, due to resource constraints, the evaluation of no reflow (NR) was based solely on coronary angiography TIMI flow grading and MBG, without utilizing more advanced tools such as cardiac magnetic resonance imaging (MRI) and myocardial echocardiography, which might introduce some degree of subjective interference.

## Conclusions

In summary, this study conducted a retrospective analysis of STEMI patients treated at our hospital over the past two years to explore the factors influencing post-PPCI myocardial perfusion and evaluate the effects of different intervention strategies on myocardial perfusion improvement. The study identified age, time from onset to balloon expansion, peak serum cTnI levels, extent of coronary artery disease, and balloon expansion pressure as independent risk factors influencing post-PPCI myocardial perfusion in STEMI patients. Compared to thrombus aspiration alone, the local intracoronary injection of nicorandil through the thrombus aspiration catheter did not significantly affect hemodynamics but further improved myocardial tissue perfusion and reduced cardiovascular events.
